# The association of dietary diabetes risk reduction score and its components with risk of metabolic syndrome incident in Tehranian adults

**DOI:** 10.1186/s12902-021-00872-w

**Published:** 2021-10-20

**Authors:** Parvin Mirmiran, Hossein Farhadnejad, Farshad Teymoori, Golaleh Asghari, Karim Parastouei, Fereidoun Azizi

**Affiliations:** 1 Nutrition and Endocrine Research Center, Research Institute for Endocrine Sciences, Shahid Beheshti University of Medical Sciences, Tehran, Iran; 2 Department of Nutrition, School of Public Health, Iran University of Medical Sciences, Tehran, Iran; 3 Health Research Center, Life Style Institute, Baqiyatallah University of Medical Sciences, Tehran, Iran; 4 Endocrine Research Center, Research Institute for Endocrine Sciences, Shahid Beheshti University of Medical Sciences, Tehran, Iran

**Keywords:** Dietary diabetes risk reduction score, Metabolic syndrome, Iranian adults

## Abstract

**Background:**

Evidence of possible beneficial effects of dietary diabetes risk reduction score (DDRRS) on reducing the risk of various chronic diseases such as metabolic syndrome (MetS) are limited. This is a prospective, population-based cohort study, which aimed to investigate the relationship of the DDRRS and its components with MetS incident in Iranian adults.

**Methods:**

Individuals without MetS (*n*=3561) were recruited from participants of the Tehran Lipid and Glucose Study (2009-2011) and followed for a mean of 6.01 years. A validated food frequency questionnaire was used to determine the DDRRS using based on eight components, including higher intakes of cereal fiber, nuts, coffee, and polyunsaturated: saturated fat ratio and lower intakes of red or processed meats, sugar-sweetened beverages, trans fatty acids, and low glycemic index. We used the multivariable logistic regression analysis to determine the odds ratio (ORs) and 95 % confidence interval (CI) of MetS across the tertiles of DDRRS.

**Results:**

The mean (SD) age of individuals was 38.1(12.6) years at baseline. Median (25-75 interquartile range) DDRRS for all participants was 20(18-22). During the study follow-up, 682(19.1 %) new cases of MetS were reported. Based on the age and sex-adjusted model, participants in highest tertile of DDRRS had lower risk of MetS in compared with the lowest one (OR=0.64;95 %CI:0.52-0.79, P for trend=0.001). In the multivariable adjusted model, after adjustment for all possible confounding variables, the risk of MetS is decreased across tertiles of DDRRS (OR=0.60;95 %CI:0.48-0.75, P for trend=0.001). Also, higher scores of some DDRRS components including red and processed meat, sugar sweetened beverages, and coffee were related to decreased incidence of MetS.

**Conclusions:**

The results of this study revealed that greater adherence to DDRRS can be associated with decreased risk of MetS in Iranian adult.

## Background

Metabolic syndrome (MetS) is a clustering of metabolic risk factors, including central obesity, hypertriglyceridemia, low high-density lipoprotein cholesterol (HDL-C), elevated blood pressure, and hyperglycemia [1], which can increase the risk of type 2 diabetes, cardiovascular disease (CVD), cancer, and all-cause mortality [2, 3]. Insulin resistance and dysregulation of lipid metabolism playing a key role in the pathogenesis of MetS [4, 5]. In addition to genetic predisposition, lifestyle factors (include smoking, physical inactivity, alcohol consumption, and especially inappropriate dietary intakes), and socioeconomic status are the main causative risk factors of MetS [2, 6–8].

Quality of the diet plays an important role in the progression of MetS [9] and the role of several nutritional factors in the incidence of MetS has been reported [10–17]. Based on single food group or nutrient-specific data in the etiology of chronic diseases dietary guidelines have some limitations, whereas whole dietary patterns are far more applicable [18, 19]. Hence investigating the association between nutritional factors in the form of a single food pattern score and risk of chronic diseases, such as MetS can provide more accurate and applicable findings.

In 2015, a study by Rhee et al. [20] computed dietary diabetes risk reduction score (DDRRS) as a healthy diet score including SSB, coffee, nuts, red and processed meats, GI, cereal fiber, the ratio of P:S, and trans fats, which indicated that higher adherence to DDRRS could decrease the risk of diabetes incident. However, to the best of our knowledge, the association of DDRRS as a single set of different dietary factors and incident MetS has not yet been assessed. Therefore, in this study, we aimed to investigate the association of DDRRS and its components with the risk of incident MetS after 6.01 years of follow-up among Tehranian adult participants.

## Methods

### Subjects

**Fig. 1:**
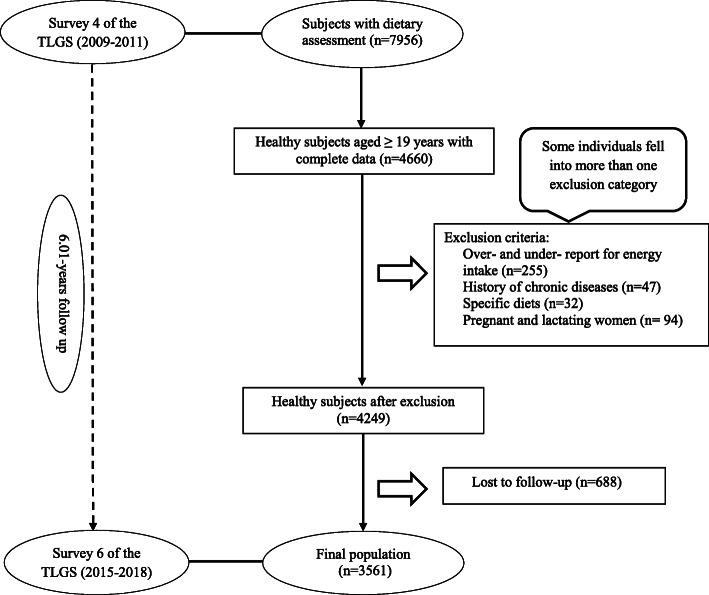
Flow chart of the Tehran Lipid and Glucose Study (TLGS) participants.

This study was performed in the framework of the Tehran Lipid and Glucose Study (TLGS), a population-based cohort study conducted to assess the risk factors for non-communicable diseases among a representative urban population of Tehran, including 15,005 participants aged ≥3 years [21]. In the fourth survey of the TLGS (2009–2011), of 12,823 participants, 7956 were randomly selected for dietary assessment. For the current study, 4660 adult men and women (aged>18 years) free of MetS at baseline, with complete baseline data, were recruited. We excluded participants who under- or over-reported energy intakes (< 800 kcal/d or > 4200 kcal/d, respectively) (*n*=255), or were on specific diets for hypertension, diabetes, or dyslipidemia (*n*=32), those with a history of myocardial infarction, cerebral vascular accident, cancer (*n*=47), and pregnant and lactating women (*n*=94); some individuals fell into more than one exclusion category. Finally, 4249 participants were followed until survey VI, for a mean period of 6.01 years from the baseline examination. After excluding the participants who left the study (*n*=688), final analyses were conducted on data of 3561 adults (Fig. 1).

### Dietary assessment

The dietary intake of participants over the previous year was assessed using a valid and reliable 168-item semi-quantitative food frequency at baseline [22]. Trained dietitians asked participants to designate their consumption frequency for each food item during the previous year on a daily, weekly, or monthly basis; portion sizes of consumed foods, reported in household measures, were then converted to grams. Since, the Iranian Food Composition Table (FCT) is incomplete and has limited data on the nutrient content of raw foods and beverages, the United States Department of Agriculture (USDA) FCT [23] was used. For national foods not listed in the USDA FCT, the Iranian FCT [24] was used.

We have determined dietary diabetes risk reduction score based on the Rhee et al. study [20] with focusing on eight components, including higher intakes of cereal fiber, nuts, coffee, and P:S ratio and lower intakes of red or processed meats, sugar-sweetened beverages, trans fatty acids, and GI. For each component, subjects were classified into quartiles according to their intake ranking, and component scores were used for cereal fiber, nuts, coffee, and P:S ratio to determine the individual’s quartiles rankings, e.g. participants in the lowest quartile were assigned 1 point, and those in the highest quartile were assigned 4 points. Scores were reversed for red or processed meats, sweetened beverages, trans fat, and GI. Therefore, individuals in the lowest quartile were given a score of 4 points, and those in the highest quartile were scored 1 point. We then summed up the points for all eight items to compute the DDRRS. The range of total DDRRS was from 8 (minimum adherence) to 32 (maximal adherence).

### Physical activity assessment

The physical activity levels of participants were determined using a modifiable activity questionnaire (MAQ), previously modified and validated among Iranians [25]. Participants were asked to report and identify the frequency and time spent on activities of light, moderate, hard, and very hard intensity during the past 12 months, according to a list of common activities of daily life; physical activity levels were reported as metabolic equivalent hours per week (MET-h/wk).

### Demographic, anthropometric, and lifestyle measures

Data on demographic, anthropometric, and biochemical variables were assessed at baseline (2006–2008). Trained interviewers used pretested questionnaires to collect information, including demographic data, medical history, medications, and smoking habits. We measured the weight of participants to the nearest 100 g using digital scales with minimal clothing and without shoes. Height was measured to the nearest 0.5 cm using a tape meter, in a standing position without shoes. Body mass index (BMI) was calculated as weight (kg) divided by the square of the height (m^2^). Blood pressure of each participant was measured twice on the right arm with a minimum interval of 30 s via a standardized mercury sphygmomanometer with an accuracy of 2 mmHg after a 15-minute rest sitting on a chair; the mean of the two measurements was considered to be the blood pressure of the participant.

### Biochemical measures

A blood sample was taken after 12-14 h of overnight fasting in a sitting position according to the standard protocol and centrifuged within 30-45 min of collection. All blood analyses were performed at the TLGS research laboratory on the day of blood collection. The samples were analyzed using the Selectra 2 auto-analyzer (Vital Scientific, Spankeren, The Netherlands). An enzymatic colorimetric method with glucose oxidase was used to determine fasting plasma glucose (FPG). Both inter-and intra-assay coefficient variations were 2.2 % for FPG. Triglyceride (TGs) level was measured using an enzymatic colorimetric analysis with glycerol phosphate oxidase. Total cholesterol (TC) was measured with cholesterol esterase and cholesterol oxidase, using the enzymatic colorimetric method. High-density lipoprotein cholesterol was measured after precipitation of the apolipoprotein B-containing lipoproteins with phosphotungistic acid. Analyses were performed using commercial kits (Pars Azmoon Inc., Tehran, Iran) and a Selectra 2 auto-analyzer (Vital Scientific, Spankeren, Netherlands). Inter-assay and intra-assay coefficients of variations were 1.6 % and 0.6 % for TGs, 2 % and 0.5 % for HDL-C, and 2 % and 0.5 % for TC, respectively. Low-density lipoprotein cholesterol (LDL-C) was calculated from the serum TC, TGs, and HDL-C concentrations and expressed in mg/dl using the Friedewald formula.

### Definitions

MetS was defined according to the joint interim statement as the presence of any 3 of 5 following factors [26]: (1) abdominal obesity as WC≥95 cm for both genders, according to the new cutoff points of WC for Iranian Adults [27]; (2) FPG≥ 100 mg/dl or drug treatment; (3) fasting TGs≥ 150 mg/dl or drug treatment; (4) fasting HDL-C<50 mg/dl for women and <40 mg/dl for men or drug treatment; and (5) high BP was defined as SBP≥130 mmHg, DBP≥85 mmHg, or antihypertensive drug treatment.

### Statistical analysis

**Fig. 2:**
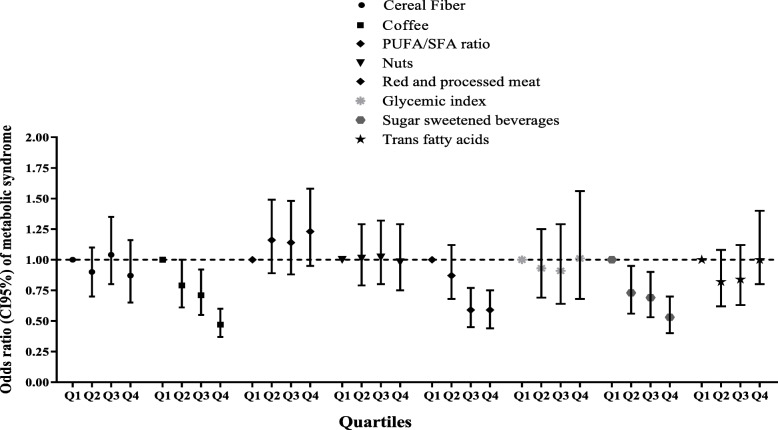
Odds ratios and 95 % CIs of incident metabolic syndrome according to quartiles of the DDRRS components scores among Tehranian adults.

The Statistical Package for Social Sciences (Version 15.0; SPSS, Chicago, IL) was used to perform all analyzes. The normality of the variables was checked using histogram charts and Kolmogorov–Smirnov test. Participants were categorized according to tertiles of DDRRS cut-points; baseline characteristics of the participants are presented as the mean ±SD or median (25-75 interquartile) for continuous variables and percentages for categorical variables. Linear regression and Chi-square were used to test the trends of continuous and categorical variables across tertiles of DDRRS, respectively. Multivariable logistic regression models were used with MetS (and its components) as the dependent variable and DDRRS as an independent variable to estimate the risk of 6.01 years incident outcomes and the odds ratio (ORs) and 95 % confidence intervals (CIs) were reported. The first tertile of DDRRS is considered as the reference category. Potential confounders were sex, age, BMI, physical activity, smoking, and daily energy intake. We also assessed the association each DDRRS components with risk of MetS incident, independently (Fig. 2). For scoring each positive component of DDRRS, subjects were classified into quartiles according to their intake ranking, and component scores were used for cereal fiber, nuts, coffee, and P:S ratio to determine the individual’s quartiles rankings, e.g. participants in the lowest quartile were assigned 1 point, and those in the highest quartile were assigned 4 points. Scores were reversed for negative components including red or processed meats, sweetened beverages, trans fat, and GI. Therefore, individuals in the lowest quartile were given a score of 4 points, and those in the highest quartile were scored 1 point. Next, individuals were categorized based on score of each component DDRRS and finally, the odds of MetS were determined across quartiles of each DDRRS components score. P-values <0.05 were considered to be statistically significant.

## Results

The mean age of participants (38.8 % male) was 38.1±12.6 years at baseline. During an average of 6.01 years of follow-up, 682 (19.1 %) new cases of MetS were identified. The median (25-75 interquartile range) of the DDRRS for all participants was 20 (18-22).

Baseline socio-demographic and biochemical characteristics of the study population across tertiles of DDRRS are reported in Table 1. Compared to the lowest tertile, participants in the highest tertile of DDRRS were more likely to be older, low smoked, and had higher levels of TC, LDL-C, HDL-C, FPG, and had lower BMI and physical activity level (*P*<0.05). No significant differences in WC, TGs, SBP, and DBP were observed in participants according to tertiles of DDRRS.

**Table 1:** Baseline lifestyle and clinical characteristics of subjects according to tertiles of dietary diabetes risk reduction score: Tehran Lipid and Glucose Study

	Dietary diabetes risk reduction score tertiles			P for trend
	Tertile 1 *n*=1429	Tertile 2 *n*=916	Tertile 3 *n*=1216	
Age (years)	36.16 ± 12.43	37.75 ± 12.16	40.79 ± 12.82	<0.001
Women (%)	58.8	61.9	63.4	0.014
Body mass index (kg/m^2^)	26.12 ± 4.15	26.13 ± 4.21	25.66 ± 4.20	0.007
Waist Circumference (cm)	88.43 ± 10.39	88.40 ± 10.52	87.68 ± 11.17	0.089
Current smoker (%)	10.2	9.0	7.7	0.025
Physical activity (MET h/week)	75.47 ± 58.12	72.09 ± 54.89	68.81 ± 50.71	0.002
High Density Lipoprotein–Cholesterol (mg /dl)	49.98 ± 10.88	50.20 ± 11.33	51.00 ± 11.35	0.021
Low Density Lipoprotein–Cholesterol(mg /dl)	107.32 ± 32.11	107.80 ± 30.14	111.82 ± 32.94	0.001
Triglycerides (mg /dl)	112.89 ± 65.58	109.61 ± 61.24	111.24 ± 55.55	0.553
Systolic blood pressure (mmHg)	108.42 ± 13.36	107.39 ± 11.89	109.32 ± 13.83	0.055
Diastolic blood pressure (mmHg)	73.12 ± 9.42	72.31 ± 9.00	73.34 ± 9.72	0.462
Fasting plasma glucose (mg /dl)	91.00 ± 8.10	92.40 ± 16.82	92.37 ± 15.66	0.009
Total cholesterol (mg /dl)	179.43 ± 36.15	179.68 ± 34.36	184.78 ± 38.00	<0.001

Data are represented as mean ± SD for continuous variables and percent for categorical variables. Linear regression and Chi-square analyses were used for test of trend continuous variables and categorical variables according to the tertiles of dietary diabetes risk reduction score, respectively

Baseline dietary intakes of participants across tertiles of DDRRS score are presented in Table 2. Individuals in the highest tertile of DDRRS had higher intakes of cereal fiber, PUFA: SFA ratio, nuts, and coffee, but lower intakes of red and processed meat, sugar-sweetened beverages, and trans fatty acids. Dietary intakes of total energy, protein, carbohydrates, total dietary fiber, vitamin C, potassium, magnesium, and calcium were significantly increased across DDRRS tertiles (*P*<0.001), whereas intakes of total fat, sucrose, and GL level were decreased (*P*< 0.001). There were no significant differences in any other nutritional measures across tertiles of DDRRS.

**Table 2:** Dietary intakes of study participants according to the tertiles of dietary diabetes risk reduction score

	Dietary diabetes risk reduction score tertiles			P for trend
	Tertile 1 *n*=1429	Tertile 2 *n*=916	Tertile 3 *n*=1216	
Dietary diabetes risk reduction score	17.16 ± 1.70	20.48 ± 0.50	23.81 ± 1.73	
**DDRRS components**				
Cereal fiber (g)	13.5 ± 28.9	14.1 ± 25.2	19.3 ± 35.8	<0.001
Coffee (cup)	0.38 ± 1.43	0.55 ± 1.41	0.78 ± 2.58	<0.001
PUFA/SFA ratio	0.57 ± 0.21	0.65 ± 0.24	0.70 ± 0.25	<0.001
Nuts (serving per week)	2.08 ± 4.11	2.35 ± 3.68	3.08 ± 9.46	<0.001
Red and processed meat (serving per day)	1.12 ± 0.87	0.70 ± 0.51	0.47 ± 0.37	<0.001
Glycemic index	228.3 ± 75.7	195.1 ± 74.8	174.1 ± 62.5	<0.001
Sugar sweetened beverages (serving per week)	5.35 ± 6.74	2.74 ± 3.60	1.32 ± 2.25	<0.001
Trans fatty acids (% energy)	4.32 ± 2.74	3.43 ± 2.88	2.09 ± 2.05	<0.001
**Other nutritional factors**				
Total energy intake (kcal/day)	2755 ± 785	2396 ± 792	2157 ± 686	<0.001
Protein (% energy)	14.45 ± 3.34	14.68 ± 2.84	15.65 ± 11.49	<0.001
Carbohydrate (% energy)	57.44 ± 6.66	57.97 ± 6.80	60.12 ± 11.84	<0.001
Total fat (% energy)	31.39 ± 6.15	30.75 ± 6.65	29.59 ± 5.71	0.001
Saturated fat (% energy)	10.81 ± 3.02	10.06 ± 2.93	9.77 ± 5.67	0.193
Monounsaturated fat (% energy)	10.47 ± 2.65	10.24 ± 2.76	10.32 ± 5.70	0.935
Polyunsaturated fat (% energy)	5.92 ± 1.82	6.20 ± 2.17	6.78 ± 5.71	0.333
Sucrose g/1000 kcal)	36.58 ± 28.55	31.56 ± 26.24	30.87 ± 21.82	<0.001
Dietary fiber (g/1000 kcal)	17.89 ± 6.40	19.34 ± 6.44	21.89 ± 9.09	<0.001
Vitamin C (mg/1000 kcal)	70.72 ± 41.65	73.84 ± 42.26	78.79 ± 50.29	<0.001
Potassium (mg/1000 kcal)	1836 ± 510	1928 ± 498	2054 ± 573	<0.001
Calcium (mg/1000 kcal)	599.4 ± 196.0	618.6 ± 191.2	637.7 ± 213.7	<0.001
Magnesium (mg/1000 kcal)	179.3 ± 35.6	192.3 ± 35.0	214.0 ± 44.6	<0.001

Data are represented as mean ± SD for continuous variables or percent for categorical variables. Linear regression was used for test of trend continuous variables according to the tertiles of dietary diabetes risk reduction score

Polyunsaturated fatty acids (PUFA) to saturated fatty acids (SFA) ratio

The OR of MetS according to tertiles of DDRRS is shown in Table 3. In the age and sex-adjusted model, the odds of MetS were lower in the highest tertile of the DDRRS compared to the lowest one (OR=0.64; 95 %CI: 0.52–0.79, P for trend=0.001). Also, in the multivariable-adjusted model, after controlling age, sex, BMI, physical activity, smoking, education level, and daily energy intake, a negative association between the high score of DDRRS and 6-year incidence of MetS remained significant (OR=0.60; 95 %CI:0.48–0.75, P for trend=0.001).

**Table 3:** Odds ratio (95 % CI) of metabolic syndrome risk according to tertiles of dietary diabetes risk reduction score among adult participants of the TLGS

	Dietary diabetes risk reduction score tertiles			P for trend
	Tertile 1 *n*=1429	Tertile 2 *n*=916	Tertile 3 *n*=1216	
Median (IQR)	18 (16-19)	20 (20-21)	23 (22-25)	
Mean ± SD	17.16 ± 1.70	20.48 ± 0.50	23.81 ± 1.73	
Case/Total	300/1429	171/916	211/1216	
Model 1†	Ref	0.85 (0.68-1.04)	0.77 (0.63-0.94)	0.010
Model 2‡	Ref	0.80 (0.64-1.01)	0.64 (0.52-0.79)	0.001
Model 3 §	Ref	0.74 (0.59-0.93)	0.60 (0.48-0.75)	0.001

†The crude model

‡ adjusted for age and sex

§ adjusted for age, sex, smoking status, total energy intake, BMI, education level, and physical activity

The ORs and 95 % CIs of incident MetS according to quartiles of the DDRRS components scores among participants are presented in Fig. 2. After adjustment of potential confounders including age, sex, smoking status, BMI, physical activity, education level, and total energy intake, OR of MetS for individuals in the highest, compared with the lowest, scores of red and processed meat was 0.59 (95 % CI:0.44-0.75), for sugar sweetened beverages 0.53 (95 % CI:0.40-0.70), and for coffee it was 0.47 (95 % CI:0.37-0.60), P for trend<0.001). In addition, a remarkable reducing linear trend was observed across the scores of red and processed meat, sugar sweetened beverages, and coffee for the risk of incident MetS (P for trend <0.001, Fig. 2).

We also showed the ORs and 95 % CIs of MetS components risk according to tertiles of the DDRRS scores among individuals in Table 4. After adjustment of potential confounders including age, sex, smoking status, BMI, physical activity, education level, and total energy intake, the odds of central obesity (OR=0.71; 95 %CI: 0.55–0.90, P for trend=0.005) and high blood pressure (OR=0.77; 95 %CI: 0.62–0.95, P for trend=0.018) were lower in the highest tertile of the DDRRS compared to the lowest one. Also, after adjustment of potential confounders, OR (95 %CI) of high blood glucose, high TGs, and low-HDL-C for individuals in the highest, compared with the lowest, score of DDRRS was 0.85 (95 % CI:0.67-1.07), 0.84 (95 % CI:0.70-1.02), and 0.94 (95 % CI:0.79-1.11), respectively, (P for trend>0.05).

**Table 4:** Odds ratio (95 % CI)* of metabolic syndrome components risk according to tertiles of dietary diabetes risk reduction score among adult participants of the TLGS

Metabolic syndrome components	Dietary diabetes risk reduction score tertiles			*P* for trend
	Tertile 1 *n*=1429	Tertile 2 *n*=916	Tertile 3 *n*=1216	
Central obesity	Ref	0.74 (0.59-0.94)	0.71 (0.55-0.90)	0.005
High blood sugar	Ref	0.98 (0.77-1.25)	0.85 (0.67-1.07)	0.179
Elevated blood pressure	Ref	0.82 (0.65-1.03)	0.77 (0.62-0.95)	0.018
High triglycerides	Ref	0.87 (0.71-1.06)	0.84 (0.70-1.02)	0.080
Low HDL-C	Ref	0.97 (0.81-1.15)	0.94 (0.79-1.11)	0.472

*adjusted for age, sex, smoking status, total energy intake, BMI, education level, and physical activity

*HDL-C* high density lipoprotein – cholesterol

## Discussion

In the current study, we investigated the association of the DDRRS and risk of MetS in the framework of longitudinal population-based study after a 6.01-year follow-up. Findings indicated that greater adherence to the DDRRS is associated with decreased risk of MetS independent of confounding variables including age, sex, smoking status, BMI, physical activity, educational level, and total energy intake. Also, higher scores of some DDRRS components including red and processed meat, sugar sweetened beverages, and coffee were related to decreased risk of MetS incident. Furthermore, our results report a negative association between DDRRS and risk of some MetS components including central obesity and high blood pressure.

To the best of our knowledge, evidence on the association between DDRRS and risk of chronic diseases is limited to the cohort study on the American population, indicating the protective role of DDRRS on the development of diabetes [20]. Our findings regarding the inverse association between DDRRS and incident MetS are consistent with the finding of the Rhee et al. study, which reported that the higher adherence to DDRRS was associated with decreased risk of type 2 diabetes in all racial and ethnic groups by more than 30 % [20]. Also, although there is no study on the association between the DDRRS-style diet and risk of MetS, our study results on the healthy characteristics of the DDRRS are comparable with beneficial effects of healthy dietary indices such as DASH and Mediterranean diet in reducing the risk of MetS. In agreement with the results of our study, Asghari et al. study has revealed that higher adherence to the DASH-style diet rich in nuts, whole grains, fruits, vegetables, and legumes with lower intakes of carbonated beverages and red meat decreased the risk of MetS [28]. Also, higher intakes of healthy food groups including whole grains, monounsaturated fat, plant proteins, seafood, fruits, and vegetables, based on the Mediterranean diet significantly have been associated with the lower risk of MetS [29].

The beneficial effect of DDRRS on decreasing the risk of MetS could be partly attributed to the inclusion of nuts, coffee, unsaturated fatty acids, and cereal fibers as positive, and red and processed meat, SSB, trans fatty acid, and high GL as negative components [10–17]. Previous reports have shown that higher intakes of each positive component of DDRRS including nuts, coffee, unsaturated fatty acids, and cereal fibers can play an important role in ameliorating the burden of MetS. Strong evidence indicates that high content of fiber in DDRRS along with low dietary GI may decrease the risk of incident MetS by improving metabolic components including glycemic control, insulin resistance, dyslipidemia, obesity, and blood pressure [15]. The mechanisms of dietary fiber on MetS components are suggested to be related to the reduction in nutrients absorption rate, appetite suppression, regulation of energy homeostasis, altering the gut microbiota, modulating inflammatory cytokines and endothelial dysfunction, regulating hormonal, and improving glucose homeostasis [15, 30, 31]. Also, the greater adherence to a high GI and GL diet is associated with the higher risk of developing MetS and central obesity, indicating an adverse effect of high-GL dietary pattern on the MetS [16]. Furthermore, it has been reported that high intakes of nuts and coffee is related to a more than 10 % reduction in the risk of MetS [14, 32]. The antioxidant components contained in nuts and coffee, including vitamin E, niacin, potassium and magnesium, fiber, MUFA, PUFA, polyphenols, phytosterols, and other dietary antioxidants suggested its protective role in MetS via the effect on inflammation and oxidative status, endothelial function, insulin resistance, and insulin secretion [14, 17, 32].

Lower consumption of negative components of DDRRS, including red and processed meat, SSB, the trans fatty acid is vital for the prevention of MetS [10–12]. A meta-analysis revealed that greater intakes of total, red, and processed meat are associated with increased risk of MetS by 14 %, 33 %, and 35 %, respectively [11]. Red and processed meats contain a high amount of total fat, saturated fat, and heme-iron. Higher SFA intake might increase MetS risk by increasing the development of obesity, hyperinsulinemia, and hyperglycemia. High levels of inflammatory mediators including C-reactive protein, increased nitrosamines production, and promotion of oxidative stress in individuals with high red and processed meat consumption might be the main reasons for the increased risk of MetS [11]. Also, high intake of TFA can cause MetS through the adverse effect on circulating lipid levels, triggering systemic inflammation, inducing endothelial dysfunction, increase of visceral adiposity, body weight, and insulin resistance [10]. Sugar-sweetened beverages have high added sugar content, low satiety potential, and incomplete compensatory reduction in energy intake, which leads to positive energy balance, so the high consumption of SSBs can increase the risk of MetS, because of their contribution to weight gain [12]. Overall, according to the components of DDRRS, this dietary score is rich in antioxidant vitamins and minerals, and phenolic compounds, and has a high unsaturated fatty acid content that mediates its effects on metabolic abnormality through possible mechanisms including anti-inflammatory, anti-oxidant, and anti-atherogenic properties, decreasing visceral adiposity and improving hyperglycemia and hyperinsulinemia.

In general, individuals with higher adherence to DDRRS score tend to have higher intakes of plant protein and plant fatty acids and lower intakes of dietary TFA and animal fats. Also, a diet with a higher score of DDRRS is a low GI dietary pattern rich in fiber and phytochemicals and poor in simple sugar. Therefore, a dietary pattern with DDRRS characteristics may be associated with a reduced risk of cardiometabolic risk factors such as insulin resistance, glucose intolerance, dyslipidemia, weight gain, and elevated blood pressure.

The current study has several important strengths. To the best of our knowledge, this is the first study with a prospective design to investigate the association of DDRRS with the risk of MetS, in a relatively large sample with a long-term follow-up. The use of valid and reliable food-frequency and physical activity questionnaires were important strengths of this study. This study however has its limitations. First, although similar to epidemiological studies, in the current study valid questionnaires were used for dietary and physical activity assessment, some measurement errors are inevitable. Also, although major confounding variables were adjusted in our models, there may still be residual or unmeasured confounders the effects of which cannot be ruled out.

## Conclusions

The results of the current study showed that greater adherence to DDRRS are associated with decreased risk of MetS. This is an important finding since it can help to define a dietary pattern that is easily adhered to by the public to prevent the growing poor health outcomes such as metabolic abnormalities.

## Data Availability

The datasets analyzed in the current study are available from the corresponding author on reasonable request.

## Abbreviations

BMI: Body mass index
CI: confidence interval
CVD: cardiovascular disease
DBP: diastolic blood pressure
DDRRS: dietary diabetes risk reduction score
FCT: Food Composition Table
FFQ: food frequency questionnaire
FPG: Fasting plasma glucose
GI: glycemic index
HDL-C: high-density lipoprotein cholesterol
MAQ: modifiable activity questionnaire
MET-h/wk: metabolic equivalent hours per week
MetS: metabolic syndrome
OR: odds ratio
TFA: trans fatty acids
P: S:polyunsaturated to saturated fats
PUFA: poly unsaturated fatty acids
SFA: saturated fatty acids
SSB: sugar sweetened beverages
TC: Total cholesterol
TGs: Triglyceride
TLGS: Tehran Lipid and Glucose Study
SBP: systolic blood pressure
SPSS: Statistical Package for Social Sciences program
